# A Rodent Model of Chikungunya Virus Infection in RAG1 ^-/-^ Mice, with Features of Persistence, for Vaccine Safety Evaluation

**DOI:** 10.1371/journal.pntd.0003800

**Published:** 2015-06-26

**Authors:** Robert L. Seymour, A. Paige Adams, Grace Leal, Maria D. H. Alcorn, Scott C. Weaver

**Affiliations:** Institute for Human Infections and Immunity, Sealy Center for Vaccine Development, Department of Pathology, University of Texas Medical Branch, Galveston, Texas, United States of America; The George Washington University School of Medicine and Health Sciences, UNITED STATES

## Abstract

Chikungunya virus (CHIKV) is a positive sense, single stranded RNA virus in the genus *Alphavirus*, and the etiologic agent of epidemics of severe arthralgia in Africa, Asia, Europe and, most recently, the Americas. CHIKV causes chikungunya fever (CHIK), a syndrome characterized by rash, fever, and debilitating, often chronic arthritis. In recent outbreaks, CHIKV has been recognized to manifest more neurologic signs of illness in the elderly and those with co-morbidities. The syndrome caused by CHIKV is often self-limited; however, many patients develop persistent arthralgia that can last for months or years. These characteristics make CHIKV not only important from a human health standpoint, but also from an economic standpoint. Despite its importance as a reemerging disease, there is no licensed vaccine or specific treatment to prevent CHIK. Many studies have begun to elucidate the pathogenesis of CHIKF and the mechanism of persistent arthralgia, including the role of the adaptive immune response, which is still poorly understood. In addition, the lack of an animal model for chronic infection has limited studies of CHIKV pathogenesis as well as the ability to assess the safety of vaccine candidates currently under development. To address this deficiency, we used recombination activating gene 1 (RAG1^-/-^) knockout mice, which are deficient in both T and B lymphocytes, to develop a chronic CHIKV infection model. Here, we describe this model as well as its use in evaluating the safety of a live-attenuated vaccine candidate.

## Author Summary

Chikungunya virus (CHIKV), the etiologic agent of chikungunya fever (CHIK), is a positive sense, single-stranded RNA virus in the genus *Alphavirus*. Chikungunya fever begins as a flu-like illness, which progresses to severe arthralgia and debilitating arthritis. This syndrome is often self-limited and rarely fatal; however many patients develop persistent arthralgia that can last from months to years. Currently there is no licensed vaccine or specific treatment for CHIKF, leaving current treatment as purely supportive in nature. The role of the adaptive immune system in disease course and viral persistence is still poorly understood. The lack of an animal model of persistent CHIKF has hindered the study of the role of the adaptive immune response and safety testing of vaccine candidates, which are under development. Due to the fact that the vaccine candidate would be deployed in areas where numerous individuals have an impaired adaptive immune system due to malnutrition or disease (HIV/AIDS); it is important to study the safety of the vaccine candidate in immunodeficient animals with no adaptive immune system. In this study we present an animal model of persistent CHIKV in adult mice, which lack an adaptive immune system and demonstrate the safety of a live-attenuated vaccine candidate.

## Introduction

Chikungunya virus (CHIKV) is a positive sense, single-stranded RNA virus in the genus *Alphavirus*, and the etiologic agent of many epidemics in Africa, Asia, Europe and most recently the Americas [1–6]. CHIKV causes chikungunya fever (CHIKF), a syndrome characterized by rash, fever, and debilitating arthralgia. In recent outbreaks, CHIKV has been recognized to manifest neurologic signs of illness in the young, elderly and in patients with co-morbidities [7]. The CHIKF syndrome is often self-limited; however many patients develop persistent arthralgia that can last months or years [8–10]. These characteristics make CHIKV not only important from a human health standpoint, but also from an economic standpoint. Currently, there are no licensed vaccines or specific treatments to prevent or control CHIKF.

Many studies of CHIKF pathogenesis have focused on the role of interferon and macrophages. As with many alphaviruses, type I interferon plays a critical role in the host response to CHIKV infection [11]. A deficiency in type I interferon signaling has been shown to cause CHIKV infection to become lethal in the mouse model [12, 13]. With immunocompetent mice, previous studies have reported that footpad swelling, myositis and tenosynivitis in adult C57BL/6J animals can be induced by footpad inoculation with wild-type (wt) CHIKV [14]. This research also demonstrated viral persistence, up to 21 days post-inoculation [15]. Results suggested that macrophages are involved in inducing footpad swelling and generating inflammatory lesions in this location [16]. Other studies have shown that CHIKV RNA can persist in the joint tissue of naturally infected humans [17] as well as experimentally infected non-human primates [18]. In mice and humans, there appears to be an overlap of up- and down-regulated genes during the arthralgic manifestations of CHIKV infection and rheumatoid arthritis, although these changes do not appear to be identical in the two diseases [19]. This raises the possibility of a derangement in the adaptive immune response to CHIKV in persons with persistent symptoms. However, the mechanism of arthralgia and persistence remain poorly understood.

Because of the focus on type I interferon and macrophages, the role of the adaptive immune response in CHIK pathogenesis has garnered little attention, especially in the mouse model. However, some recent studies have begun to elucidate the role of the adaptive immune response [20, 21] by demonstrating a possible role for CD4+ T cells in the pathogenesis of CHIK and persistence in young mice with deficits of the adaptive immune response.

In addition to understanding fundamental aspects of CHIK pathogenesis and its persistence, murine models are needed to evaluate the safety of vaccines, especially live-attenuated candidates. One such strain, 181/clone 25 [22], was tested through phase II human trials, where it proved highly immunogenic but mildly reactogenic [23]. We recently developed a live-attenuated vaccine candidate for CHIK based on the insertion of an internal ribosome entry site (IRES) sequence into the CHIKV genome. This vaccine candidate (CHIKV/IRES) is safe and immunogenic in type 1 interferon-deficient mice [12, 13] as well as in cynomolgus macaques [24]. However, due to the prevalence of HIV infection as well as immunosuppressive therapies for cancer and other chronic diseases, live-attenuated vaccines should ideally be safe in persons with compromised adaptive immune responses. In addition, malnutrition in resource-limited areas where CHIK is endemic, which can lead to immune suppression, also underscores the need to evaluate live vaccine strains in adaptive immune-deficient models.

To improve understanding of the role of the adaptive immune response in CHIK, especially in chronic arthritic manifestations, and to further evaluate the safety of our live-attenuated vaccine candidate, we used adult recombinase activating gene-1 (RAG1^-/-^) knockout mice, which are deficient in both T and B lymphocytes. We performed safety studies with the CHIKV/IRES vaccine candidate using RAG1^-/-^ mice to evaluate the dependence on adaptive immunity of control of vaccine virus replication and pathogenesis. Also, because the roles of T and B cells in acute and chronic CHIKV infection have received limited attention, we sought to determine if the clinical manifestations (i.e. footpad swelling) of murine infections by CHIKV are T cell-driven. In addition, we wanted to determine if the adaptive response is necessary for viral clearance by determining whether its absence leads to viral persistence or death, and potential sites of persistent viral replication.

## Materials and Methods

### Viruses

A wt CHIKV strain from La Reunion derived from a cDNA infectious clone was described previously [25]. The wt CHIKV as well as vaccine strain 181/clone 25 were passaged once on Vero cells. The CHIKV/IRES vaccine strain was generated by electroporation of Vero cells with RNA transcribed from a cDNA as described previously [13] and passaged once on 293 cells.

### Ethics statement

Work with infected animals was carried out in either animal biosafety level (ABSL)-2 or -3 facilities under an approved UTMB Institutional Animal Care and Use Committee (IACUC) protocol 02-09-068. All animals were cared for in accordance with the guidelines of the Committee on Care and Use of Laboratory Animals (Institute of Laboratory Animal Resources, National Research Council).

### Animal experimental design

RAG1^-/-^ mice on the C57BL/6J background were purchased from Jackson Laboratories (Bar Harbor, ME) and were 8–10 weeks of age at the time of inoculation. On day 0, RAG1^-/-^ and C57BL/6J mice were inoculated either subcutaneously (SC) or in the footpad (FP) with either wt CHIKV (3 or 5 log_10_ PFU), vaccine candidate CHIKV/IRES (3 or 5 log_10_ PFU), vaccine strain 181/clone 25 (3 or 5 log_10_ PFU), or PBS. On days 1–14 after infection, animals were weighed and FP height (in mm) was also measured. Serum was collected on days 1–7, 14, and 28 after infection to assay viremia. Two to 3 mice from each cohort were sacrificed on days 2, 4, 7, 14, 28, 42, 56, and 70 after infection for collection of tissues for histopathologic evaluation and for measuring viral load by plaque assay. Frozen (-80°C) tissues were thawed in a 10X volume of DMEM and homogenized using a TissueLyserII (Qiagen, Valenica, CA) at 25 cycles per second for 4 minutes. The homogenate was clarified by centrifugation (2500 x g for 5 min) and the supernatant was removed and stored at -80°C for plaque assay.

### Plaque assays for viremia and tissue viral load

Plaque assays were performed on Vero cells (American Type Culture Collection, Manassas, VA) in either 6 or 12 well plates as described previously [26]. Titrations were overlaid with 0.2% agarose in Dulbecco’s modified minimal essential medium (DMEM). Two or 3 days later, cells were fixed with 10% formaldehyde for at least 30 minutes and stained with crystal violet to visualize plaques. Plaque assays were performed in duplicate or triplicate depending on the amount of serum available.

### Histology

Tissues were fixed in 10% neutral buffered formalin (RICCA Chemical Co., Arlington, TX), and bone tissue was decalcified overnight using Fixative/Decalcifier (VWR International, Radnor, PA). Organs were embedded in paraffin and 5μm sections were cut for histopathological analysis. Sections for hematoxylin and eosin staining (H&E) were prepared as previously described [13].

### Viral RNA extractions and sequencing

Total RNA was extracted from 140 μl of viral stocks or 120 μl of 10% tissue homogenates using the Viral mRNA extraction kit (Qiagen, Valencia, CA) and the manufacturer's protocol. cDNA was synthesized using the SuperScriptIII First Strand kit (Invitrogen) with random hexamer primers. Then, 3 μl reverse transcription mixtures were used for PCRs, to generate overlapping amplicons covering the entire genome, with Phusion Hot Start II High-Fidelity DNA Polymerase (New England BioLabs, Ipswich, MA) and CHIKV-specific primers. (sequences are available upon request). Amplicons were purified from agarose gels with the QIAquick Gel Extraction kit (Qiagen, Valencia, CA) and used for direct sequencing.

### Statistics

Differences in animal weights and footpad swelling were analyzed using two-way ANOVA with a Tukey-Kramer post-hoc test. Significance was determined by a p-value of <0.05.

## Results

### Clinical outcome

**Fig 1:**
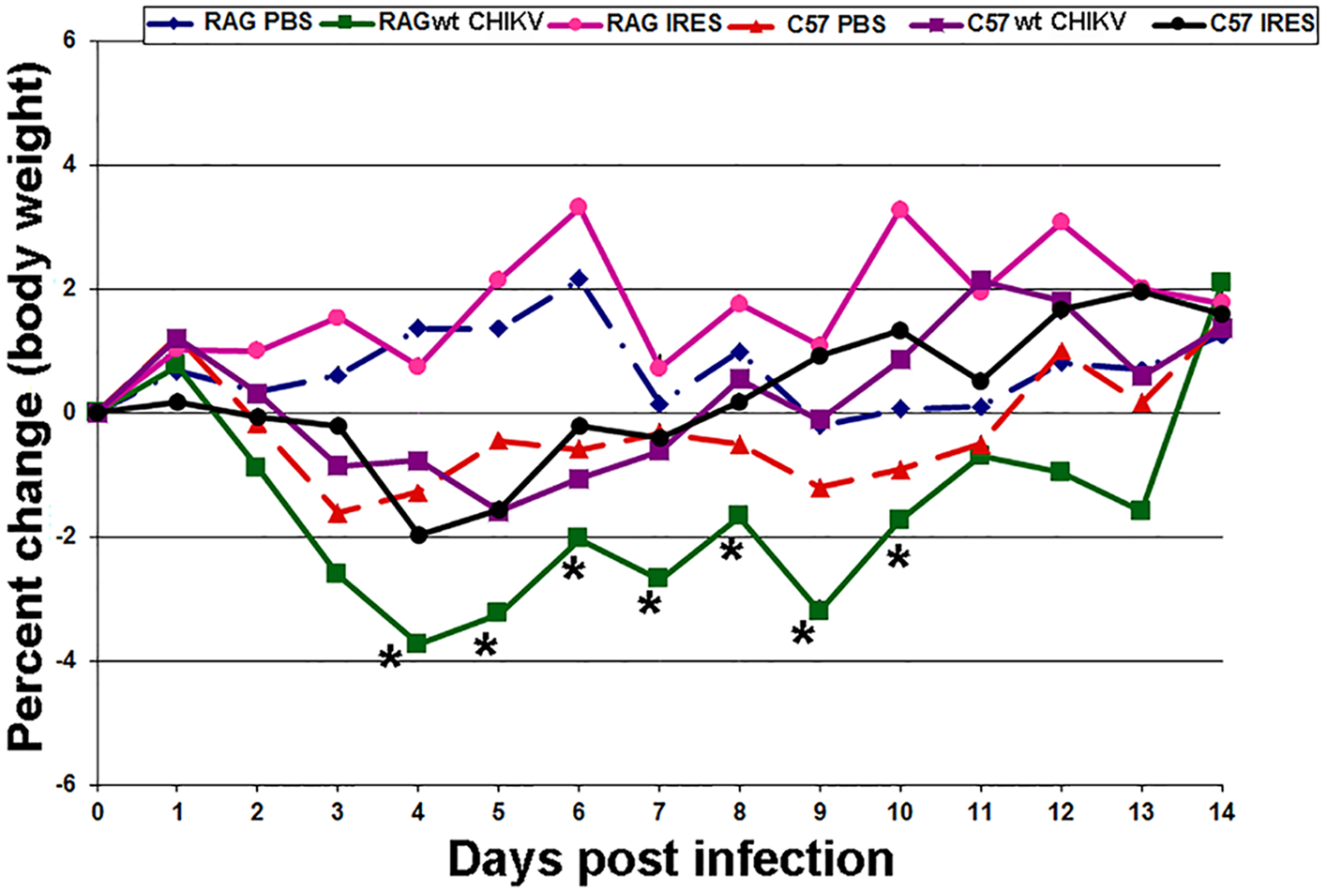
Percent change in body weight in C57BL/6J and RAG1^-/-^ mice after footpad inoculation with CHIKV/IRES or wt CHIKV (3 log_10_ PFU). There was weight loss (>3%) in RAG1^-/-^ mice infected with wt CHIKV, which gradually improved, and there was mild weight loss (<2%) in C57BL/6J mice infected with wt CHIKV and in C57BL/6J mice infected with CHIKV/IRES, which also improved. There was no weight loss in RAG1^-/-^ mice infected with CHIKV/IRES. For all mice, there were no clinical signs of illness (e.g., lethargy, ruffled fur) detected after infection. *denotes statistical significance (p<0.05).

To determine the effects of wt CHIKV, CHIKV/IRES and CHIKV vaccine strain 181/25, mice were inoculated either SC or via the FP (each route 10^3^ PFU). There were no clinical signs of illness (e.g., lethargy, ruffled fur) following SC or FP inoculation of RAG1^-/-^ or congenic C57BL/6J mice with either vaccine strain or wt CHIKV, and no change in weight was identified after SC infection regardless of the virus strain (S1 Dataset). This was in contrast to RAG1^-/-^ and C57BL/6J mice infected with wt CHIKV via the FP, which showed mild but significant weight loss compared to sham-infected controls (Fig 1). Vaccine strain 181/25 was not tested via the FP route.

### Viremia

**Fig 2:**
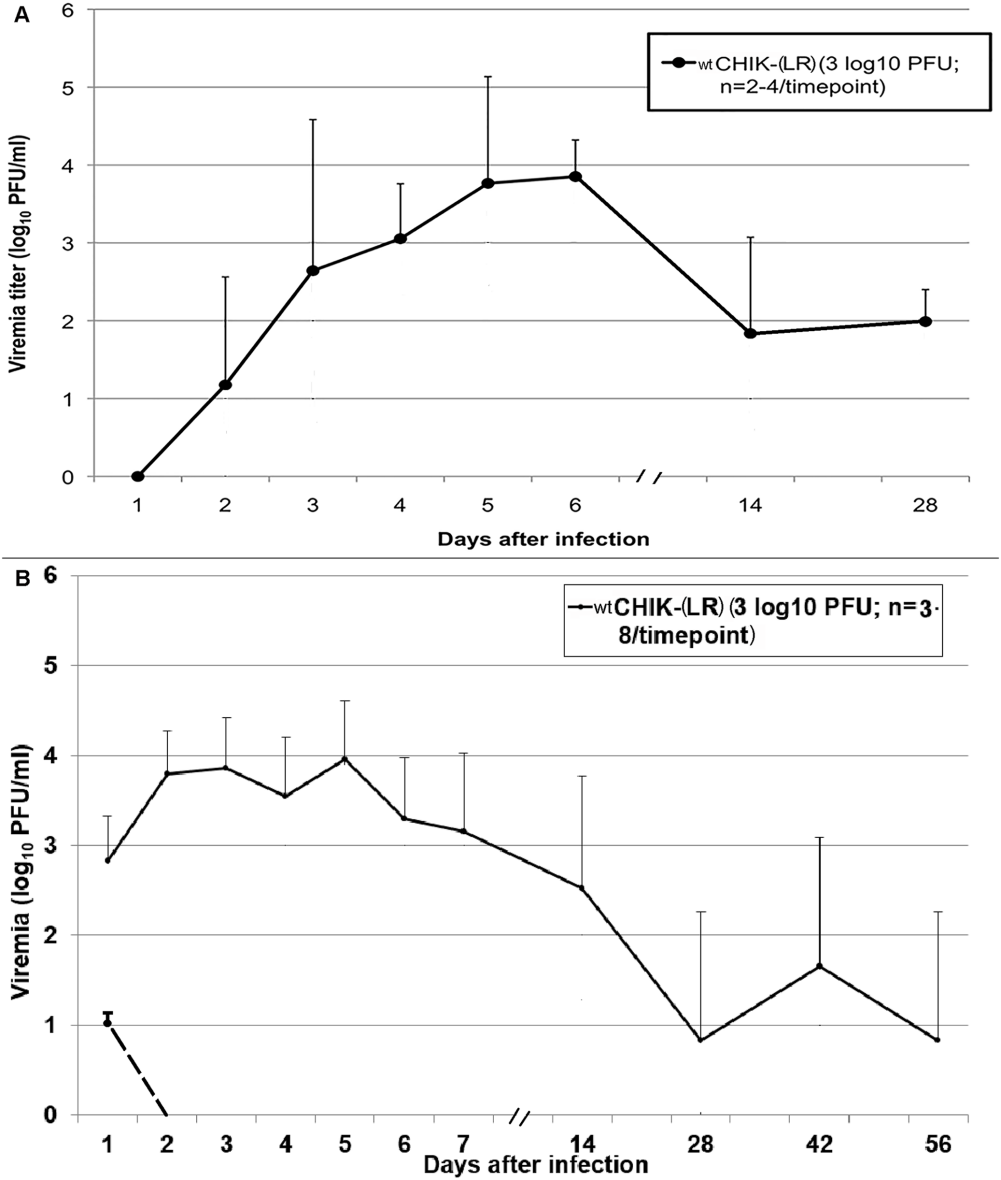
Viremia in RAG1^-/-^ mice after SC (A) and FP (B) inoculation with wt CHIKV (3 log_10_ PFU). C57BL/6J mice did not develop viremia after SC inoculation but developed a 10 PFU/ml viremia on day 1 after footpad inoculation. None of the vaccine cohorts in RAG1^-/-^ or C57BL/6J mice developed viremia (limit of detection, 10 PFU/ml). Error bars represent one standard deviation; in (A) n = 4 for days 1–14 and n = 2 for day 28; in (B) n = 7 or 8 for days 1–14 and n = 3 for days 28, 42 and 56.

Unlike C57BL/6J mice, RAG1^-/-^ mice developed persistent infection when inoculated with wt CHIKV by either route (Fig 2). Viremia reached a peak titer of about 4 log_10_ PFU/ml on days 5–6 after SC infection, gradually decreasing to 2 log_10_ PFU/ml on days 14 and 28; no viremia was detected after day 28 post-infection (Fig 2A). These findings were in contrast to the vaccine candidate strains, which never produced detectable viremia.

C57BL/6J mice inoculated via the FP with wt CHIKV showed a peak viremia of about 10 PFU/ml on day 1 post-infection (Fig 2B). RAG1^-/-^ mice again showed persistent infection as evidenced by prolonged viremia (Fig 2B). Like mice inoculated SC, they had a peak viremia of 4 log_10_ PFU/ml on day 5–6 post-infection, gradually decreasing to 2 log_10_ PFU/ml. However viremia persisted in these animals until at least day 56 post-inoculation. In contrast, the CHIKV/IRES vaccine strain never produced detectable viremia. RAG1^-/-^ mice inoculated with 5 log_10_ PFU of wt CHIKV showed a similar pattern of viremia to those inoculated with 3 log_10_ PFU (S2 Dataset).

### Viral load in tissues

To assess tissue tropism and persistence, we harvested the organs of RAG1^-/-^ and C57BL/6 mice inoculated with wt CHIKV, CHIKV/IRES or vaccine strain 181/25. Both SC and FP routes of inoculation were tested (Tables 1 and 2). Neither CHIKV/IRES nor strain 181/25 was detected in RAG1^-/-^ or C57BL/6J mice regardless of route of inoculation, and wt CHIKV was not detected in C57BL/6J mice regardless of the route. However, wt CHIKV predominantly persisted in the brain and kidney following SC inoculation of RAG1^-/-^ mice regardless of the route. Other major organs (heart, skeletal muscle) were sporadically positive for persistent virus, mainly in those mice inoculated via the FP.

**Table 1:** Viral loads in organs of RAG1^-/-^ mice following SC infection with 3 log_10_ PFU of wild-type CHIKV^a^.

Organ	Days after infection	No. of positive organs/total tested^b^	Mean viral titer ± SD (log_10_ PFU/g)^c^
Brain	28	1/2	5.6
	42	0/2	n/a
	56	0/2	n/a
	70	0/2	n/a
Kidney	28	2/2	6.1 ± 0.2
	42	1/2	7.0
	56	1/2	4.0
	70	0/2	n/a

^a^No virus was detected in the organs of mice vaccinated with either CHIKV/IRES or vaccine strain 181/25

^b^limit of detection, 10 PFU/g

^c^n/a, data not available

**Table 2:** Viral loads in organs of RAG1^-/-^ mice following footpad infection with 3 log_10_ PFU of wt CHIKV^a^.

Organ	Days after infection	No. of positive organs/total tested^b^	Mean viral titer ± SD (log_10_ PFU/g)
Kidney	28	1/3	4.79
	42	1/3	4.90
	56	1/3	2.60
Heart	42	1/3	4.48
Muscle	28	1/3	4.90

^a^The heart and skeletal muscle were not positive in any animal tested on any other time point except as above. No virus was detected in the organs of mice vaccinated with CHIKV/IRES; vaccine strain 181/25 was not tested via FP injection.

^b^limit of detection 10 PFU/ml.

### Footpad swelling

**Fig 3:**
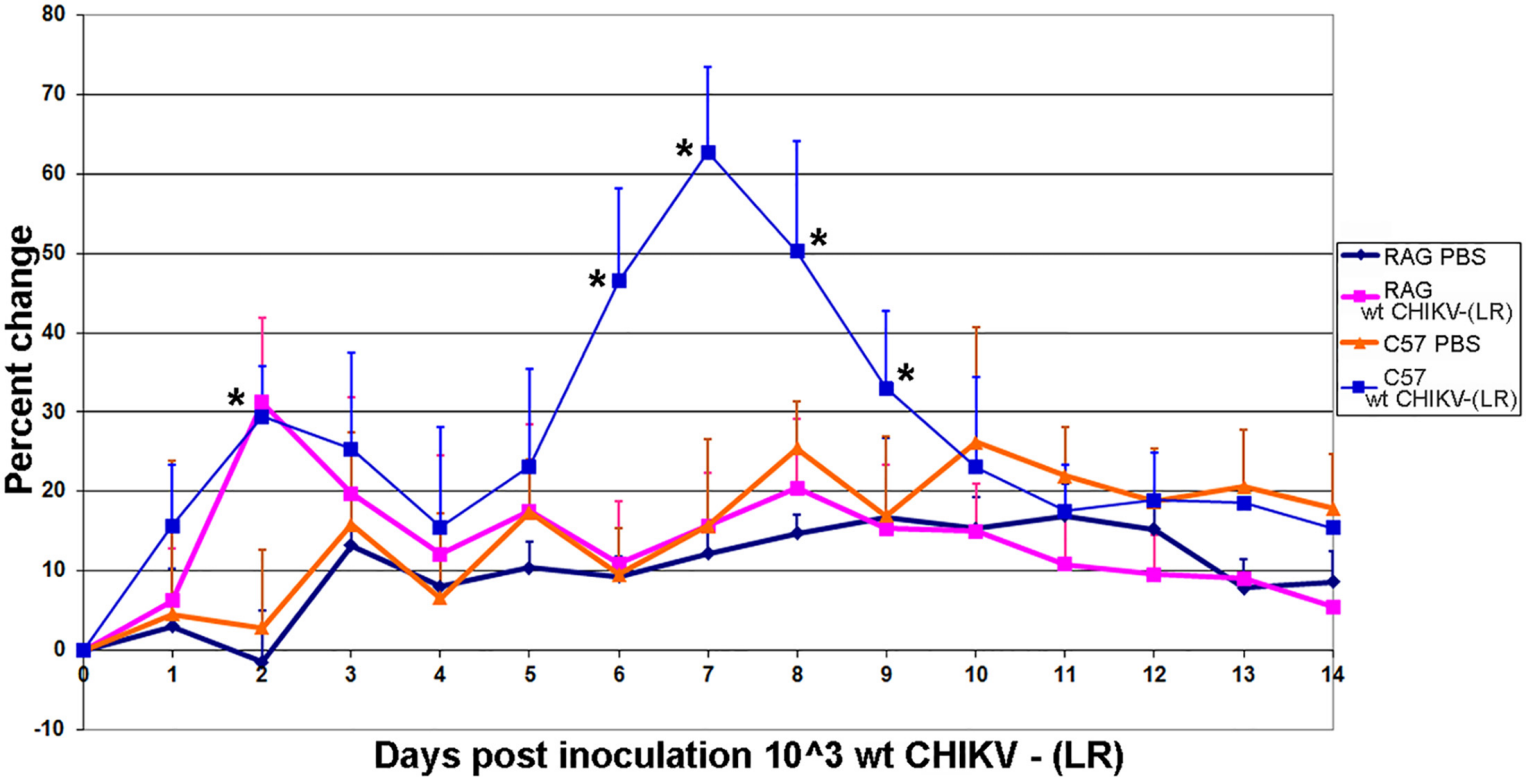
Percent change in FP swelling in C57BL/6J and RAG1^-/-^ mice following FP infection with either PBS or 3 log_10_ PFU of wt CHIKV. There was a biphasic pattern of inflammation seen in C57BL/6, but not RAG1^-/-^ mice. Error bars represent one standard deviation. *denotes statistical significance (p<0.05).

Following FP inoculation with wt CHIKV, C57BL/6J mice developed a biphasic pattern of inflammation at the injection site that was not observed with RAG1^-/-^ mice (Fig 3). C57BL/6J and RAG1^-/-^ animals showed some footpad swelling on day 2 post-inoculation, which decreased to baseline levels by day 3. C57BL/6J mice inoculated with wt CHIKV showed increased swelling at day 6, peaking at day 7, and resolving by day 10 post-inoculation. In contrast, RAG1^-/-^ mice never showed footpad swelling after day 3, and similarly, mice inoculated with vaccine strain CHIKV/IRES never exhibited footpad swelling.

### Histopathologic findings

**Fig 4:**
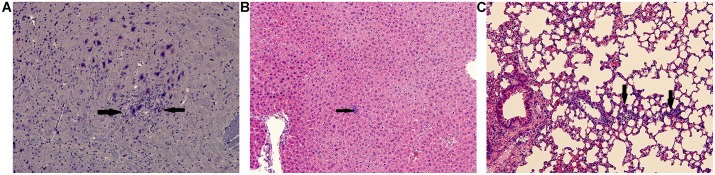
Hematoxylin and eosin staining of representative sections from RAG1^-/-^ mice inoculated with wt CHIKV. (A) Brain Day 28, (B) Liver Day 42, (C) Lung Day 42; all images were taken at 20x; scale bar is 200μm. Arrows indicate areas of inflammation.

To evaluate tissue damage following each inoculation route (SC or FP), the brain, heart, lung, kidney, spleen, stomach, intestines, liver, gonads and leg tissues (including joints) were collected on days 7, 14, 28, 42, 56 and 70 post-inoculation and evaluated histologically. Poorly formed granulomatous inflammation was identified in the brain, liver and lungs of RAG1^-/-^ mice inoculated SC with wt CHIKV on days 28–56 (Fig 4). No inflammation was identified in the leg tissues of these animals. C57BL/6J animals showed poorly formed granulomatous inflammation only in the liver (Fig 4A). The areas of granulomatous-type inflammation were identified in small foci. The inflammation in the brain of the RAG1^-/-^ mouse was small and confined to the cerebrum, with no evidence of meningitis (Fig 4B). The inflammation in the lung consisted of tiny foci (Fig 4C). Several small poorly formed granulomas were identified in the livers of RAG1^-/-^ and C57BL/6J mice, with no evidence of hepatocyte necrosis (Fig 4A). Granulomatous inflammation in the RAG1^-/-^ mice occurred only in those identified to have persistent CHIKV in either their serum or organs. Those RAG1^-/-^ mice that were negative for persistent CHIKV were histologically normal. This was observed only in mice with persistent CHIKV infection and not in those without detectable CHIKV housed in the same cages. This finding suggests that the inflammation was caused by persistent CHIKV infection and not another pathogen infecting the colony or cages of immunodeficient mice. In similar fashion, some animals (both RAG1^-/-^ and C57BL/6J) inoculated via the FP had very small granulomas in the liver only, similar to Fig 4B.

**Fig 5:**
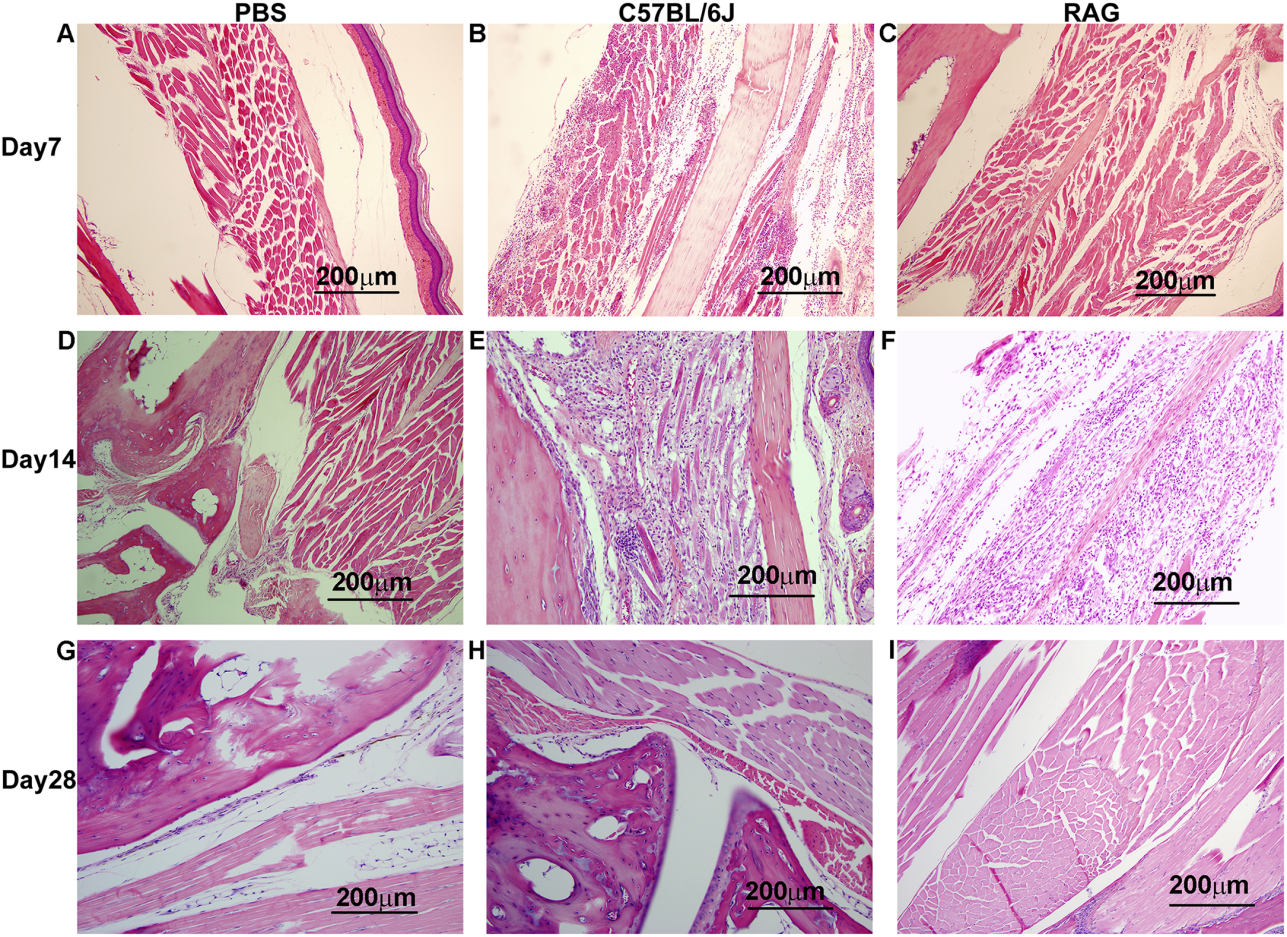
Hematoxylin and eosin staining of representative footpads from C57BL/6J or RAG1^-/-^ mice inoculated with PBS or wt CHIKV. (A) PBS Day 7, (B) C57BL/6J wt CHIKV Day 7, (C) RAG1^-/-^ wt CHIKV Day 7, (D) PBS Day 14, (E) C57BL/6J wt CHIKV Day 14, (F) RAG1^-/-^ wt CHIKV Day 14, (G) PBS Day 28, (H) C57BL/6J wt CHIKV Day 28, (I) RAG1^-/-^ wt CHIKV Day 28; all images were taken at 20x; scale bar is 200μm. Images of PBS controls were taken from RAG1^-/-^ mice.

These findings contrasted with those seen in the animals inoculated via the FP (Fig 5); following this route, C57BL/6J mice exhibited severe myositis in the inoculated leg on day 7 (Fig 5B). By day 14 post inoculation, inflammation was resolving and muscle regeneration had begun (Fig 5E); lymphocytes and neutrophils had left the lesions leaving only macrophages behind. Many of the cells present, which appeared large, were regenerating myocytes. By day 14 macrophages would be expected to be present to promote wound healing and muscle regeneration. By day 28, the lesions were healed (Fig 5H). The results in RAG1^-/-^ mice inoculated via the FP were surprising because, as noted above, no footpad swelling was noted after day 2 in these mice following FP injection with wt CHIKV (Fig 3). Based on these results and previous studies, we did not expect to observe any inflammation or tissue damage in the RAG1^-/-^ mice. At day 7 post-inoculation, no inflammation or tissue damage was noted in these animals (Fig 5C). However, at day 14 post inoculation, severe muscle damage was observed along with a mild inflammatory infiltrate. The muscle damage observed at day 14 (Fig 5F) in RAG1^-/-^ mice was comparable to damage seen in wt mice at day 7 post inoculation (Fig 5B). By day 28 the lesions were healed (Fig 5I). Sham-infected animals had no evidence of histologic damage in the FP (Fig 5A, 5D and 5G). The PBS groups from RAG1^-/-^ and C57BL/6J mice were identical.

### Sequences of viruses isolated from tissues

To determine if mutations could be involved in CHIKV persistence in RAG1^-/-^ mice, we randomly selected four tissue samples collected on days 28 and 42 for complete genome sequencing (Table 3). When compared to the sequence of the inoculum strain, one sample from a day 28 kidney sample showed an identical consensus sequence. Virus from the other three samples had mutations only in the non-structural protein (nsP) genes; the majority were synonymous, while only two were non-synonymous. However, two identical synonymous mutations in nsP3 and nsP4 occurred in two independent samples from different animals on different days, suggesting positive selection at the nucleotide level.

**Table 3:** Mutations identified in wt CHIKV recovered from persistently infected organs harvested from RAG1^-/-^ mice^a^.

Organ	Days after infection	Gene	Codon	Nucleotide change	Amino acid change
Kidney	42	NSP2	758	C➔T	
		NSP4	188	C➔T	Pro➔Ser
Kidney	42	NSP3	301	A➔G	
		NSP4	213	G➔A	
Brain	28	NSP1	369	C➔T	
		NSP3	301	A➔G	
		NSP4	213	G➔A	
		NSP4	604	A➔G (mixed population)	Ile➔Val

^a^ All samples were obtained from different animals on the days indicated.

## Discussion

Our study had three key findings: 1) the adaptive immune system is not only critical for clearance of CHIKV, but it plays a role in the inflammatory response to infection; 2) tissue damage occurs in the absence of an adaptive immune response, and; 3) the newly developed CHIKV/IRES vaccine candidate and strain 181/25 do not persist in mice, even in the absence of T/B cells. The latter point is very important when considering vaccine safety, because many people in developing countries who are exposed to CHIKV are also immunocompromised due to various conditions (e.g., HIV, malnourishment, etc.).

Viral pathogenesis research has recently focused on the type I interferon response, which is also critical in controlling many alphavirus infections, including CHIKV. Also, macrophages/monocytes are known to precipitate tissue damage in mice infected with the related Ross River alphavirus, and have been implicated in CHIKV pathogenesis in mice and humans [16, 18, 27].

The role of the adaptive immune response in the clearance and pathogenesis of CHIKV infection is also beginning to be explored using mouse models. One study implicated a role for CD4+ T cells in the pathogenesis of CHIK, and another showed persistence in mice deficient in the adaptive immune response [20, 21]. Our experiments, while confirming some findings of these previous studies, also generated contradictory findings. We found that T cells are not entirely responsible for the CHIK disease process. While T cells are involved in footpad swelling, disease, as indicated by histologic damage, occurs in the absence of T cells. This damage is delayed in RAG1^-/-^ compared to C57BL/6J mice, although the muscle damage seen by day 14 post infection is comparable in the two strains. Also, the adaptive immune system is not necessary to clear footpad disease in the adult mouse. The muscle and footpad recover in RAG1^-/-^ mice, just as in C57BL/6J mice, by day 28 post infection. The adaptive response though does appear to be necessary for complete viral clearance.

We used adult 8-10-week-old RAG1^-/-^ mice for several reasons. First, the live-attenuated CHIKV vaccine candidate CHIKV/IRES is progressing toward clinical trials and wanted to explore its safety under immunocompromised conditions because many people in CHIK-endemic regions are immunosuppressed (HIV, malnutrition). Second, weanling C57BL/6J mice, another CHIK model used in previous studies [13, 28], are highly susceptible to alphaviral disease regardless of the status of the adaptive immune system. In fact, wt mice are susceptible to CHIK at this age. However, it is well known that older mice (8–10 wks) are not nearly as susceptible to most alphaviral disease, including CHIK. In addition to this age-dependence, 8-10-week-old mice have a fully developed innate immune response compared to younger mice. Using older RAG1^-/-^ mice for our studies overcame many of these confounding factors.

Although others have found colocalized viral mRNA and tissue damage in younger RAG1^-/-^ mice and have observed tenosynovitis for extended periods of time, we did not observe these findings using adult RAG1^-/-^ mice [21]. Despite damage in the ipsilateral skeletal muscle of the leg at day 14, no infectious virus was isolated beyond day 28 post-infection in RAG1^-/-^ mice. Interestingly, the organ most consistently and persistently infected, regardless of the route of infection, was the kidney. Why the kidney is specifically targeted for persistence without apparent histologic damage remains a subject of further investigation. However, the kidney is not an unusual target for persistence, given the findings associated with West Nile virus infections in hamsters [29, 30] and humans [31].

The changes in the CHIKV viral genome sequence that we observed during persistence do not appear to be random, but instead seem to focus on the non-structural protein (nsP) genes, with identical synonymous mutations occurring in independent samples from different animals. Because synonymous mutations are not typically associated with convergence reflecting positive selection, reverse genetic experiments beyond the scope of the current study are needed to determine whether they play a role in CHIKV persistence.

Our results along with others point to an important role of the adaptive immune system in controlling persistent CHIKV infection and/or its sequelae. Despite its control of persistent infection, the adaptive immune response does not prevent acute disease or tissue damage. In fact, the presence or absence of the adaptive response does not appear to affect the amount of damage to the skeletal muscle of the injected FP; its absence only appears to delay the damage by a few days. Our data also indicate that FP swelling is not necessarily a good measure of CHIK. Although the absence of T-cells completely abrogated FP swelling, disease was still detected by histopathologic analysis. Overall, our results combined with those of previous studies of the adaptive immune response [20, 21], along with those focusing on macrophages/monocytes and interferons [9–18], indicate that each part of the immune system plays an important, yet different and sometimes overlapping role in controlling CHIKV infection and disease.

Our findings could have important implications for the treatment and understanding of CHIKV infections in humans. Individuals with HIV/AIDS may not always manifest overt signs and symptoms despite having disease. Immunocompromised individuals could be viremic for extended periods of time, like our RAG1^-/-^ mice. Though the viremia seen in RAG1^-/-^ mice is too low for mosquito transmission, more study needs to be done in individuals with HIV/AIDS to determine the levels of viremia and if sustained viremia occurs. Those with HIV/AIDS could also conceivably have the granulomatous-type inflammation seen in persistently infected RAG1^-/-^ mice. Our results of persistent kidney infection also suggest that it might be useful to test the urine of acutely infected individuals and those suffering from persistent CHIK symptoms to determine if virus is shed there.

Finally our study demonstrates the safety of the CHIKV/IRES vaccine candidate in RAG1^-/-^ mice. Previous studies have demonstrated the safety of this vaccine in mice deficient in a type I interferon response (A129) [13], which is important given that this innate defense is critical for controlling the early stages of alphavirus infections. Because this live-attenuated vaccine candidate would ideally be deployed in areas of the world where many individuals are deficient in adaptive immune responses, (i.e. HIV/AIDS and malnutrition) its safety in this population is critical. Therefore, the lack of CHIKV/IRES detection in any tissues or in serum of RAG1^-/-^ mice, as well as the lack of any signs of disease, including histologic lesions, suggests that it is safe for immunization of immunocompromised people.

## Supporting Information

S1 Dataset Percent change in body weight in C57BL/6J and RAG1^-/-^ mice after SC inoculation with CHIKV/IRES or wt CHIKV (3 log_10_ PFU). There was no statistically significant weight loss in RAG1^-/-^ or C57BL/6J mice infected with either wt CHIKV or CHIKV/IRES. For all mice, there were no clinical signs of illness (e.g., lethargy, ruffled fur) detected after infection.
(TIF)

S2 Dataset Viremia in RAG1^-/-^ mice after SC inoculation with wt CHIKV (5 log_10_ PFU). C57BL/6J mice did not develop viremia after SC inoculation. None of the vaccine cohorts in RAG1^-/-^ or C57BL/6J mice developed viremia (limit of detection, 10 PFU/ml). Error bars represent one standard deviation; n = 4 for each day.
(TIF)
