# Supplementary figures and images for: A Rodent Model of Chikungunya Virus Infection in RAG1 -/- Mice, with Features of Persistence, for Vaccine Safety Evaluation
Source: PLoS Negl Trop Dis. 2015 Jun 26;9(6):e0003800. doi: 10.1371/journal.pntd.0003800 (PMC4482609; doi:10.1371/journal.pntd.0003800)

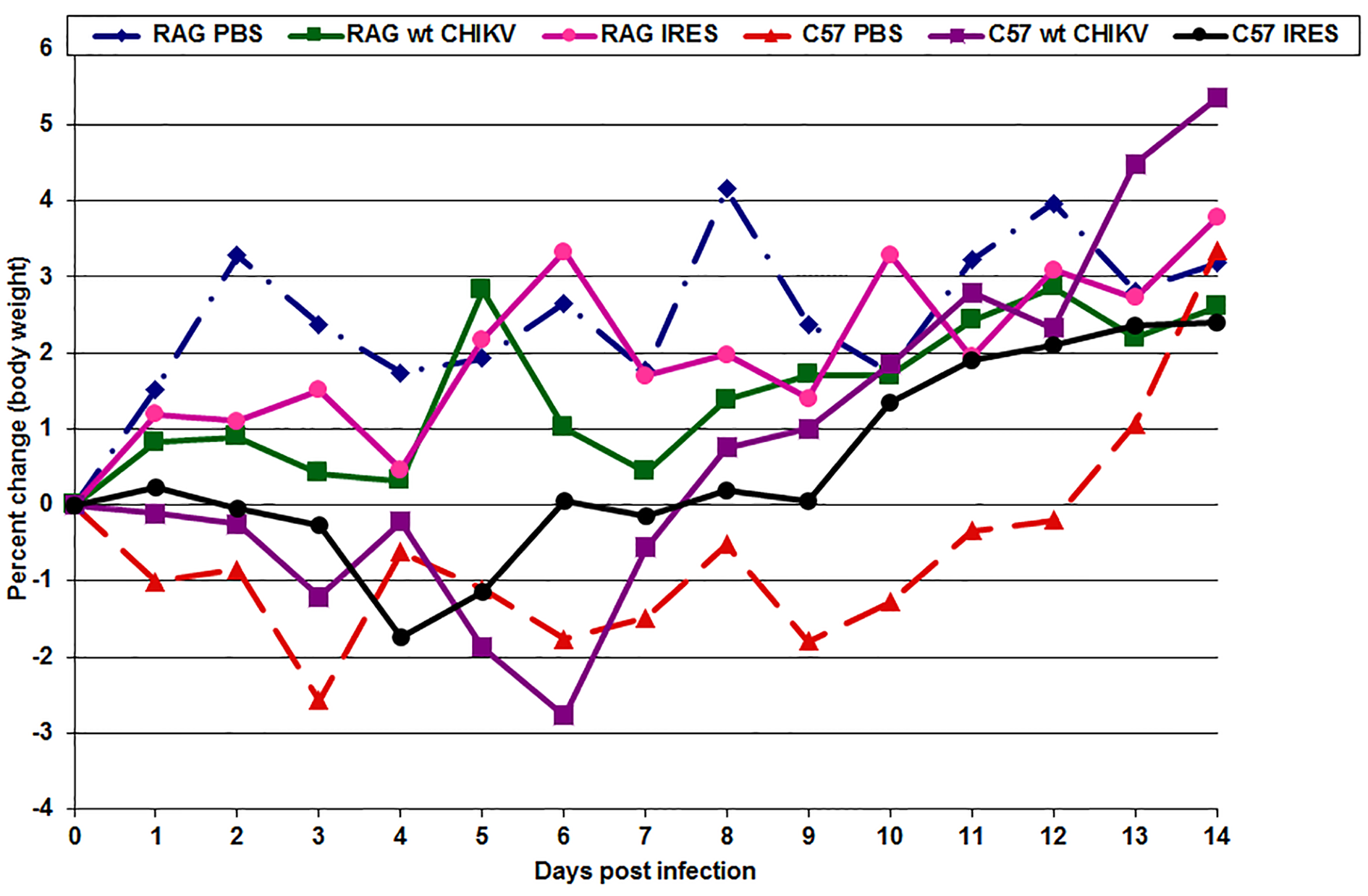

Supplement: S1 Dataset — There was no statistically significant weight loss in RAG1-/- or C57BL/6J mice infected with either wt CHIKV or CHIKV/IRES. For all mice, there were no clinical signs of illness (e.g., lethargy, ruffled fur) detected after infection. (TIF) [file pntd.0003800.s001.tif]

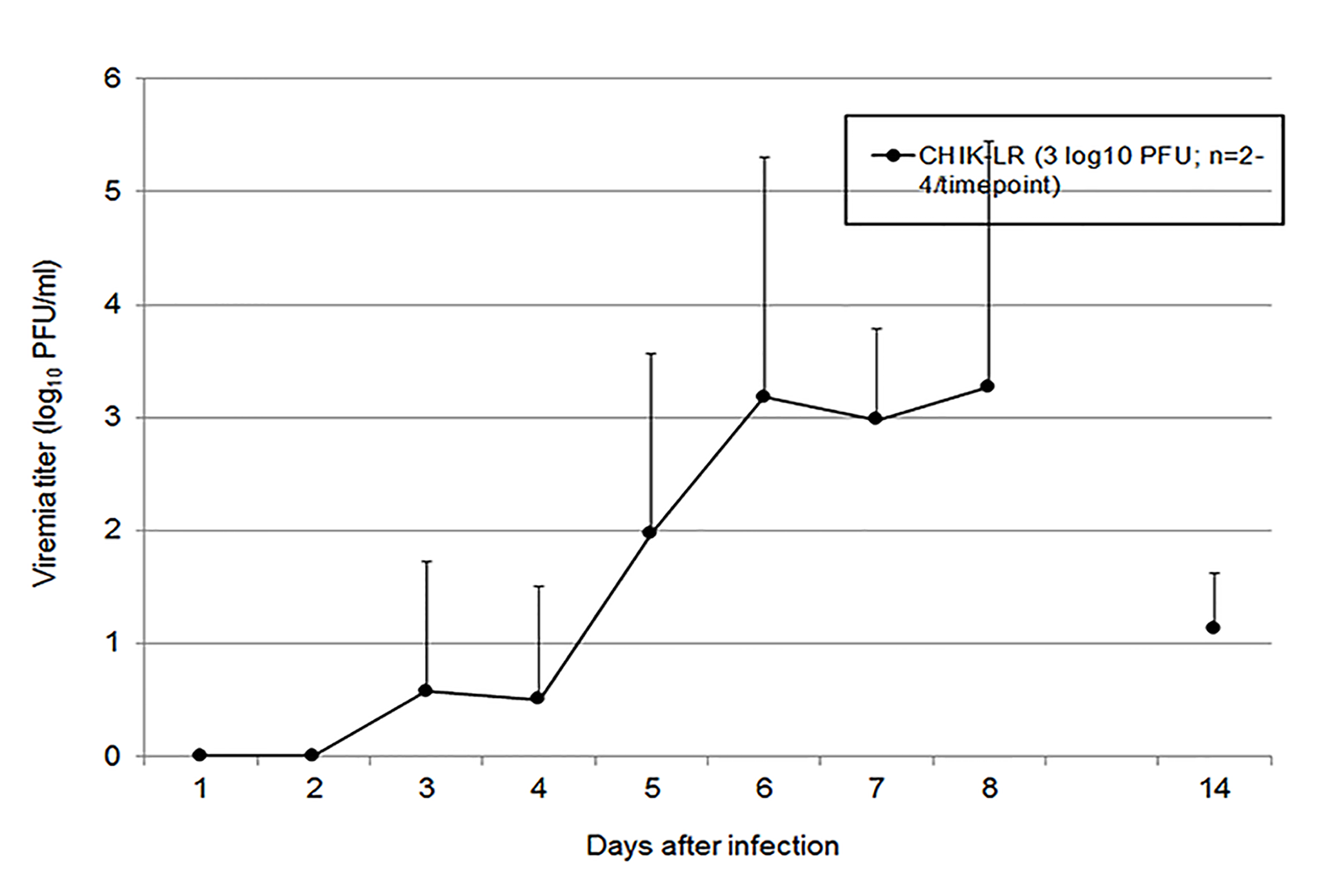

Supplement: S2 Dataset — C57BL/6J mice did not develop viremia after SC inoculation. None of the vaccine cohorts in RAG1-/- or C57BL/6J mice developed viremia (limit of detection, 10 PFU/ml). Error bars represent one standard deviation; n = 4 for each day. (TIF) [file pntd.0003800.s002.tif]
